# Short-chain fatty acids (SCFA) in infants’ plasma and corresponding mother’s milk and plasma in relation to subsequent sensitisation and atopic disease

**DOI:** 10.1016/j.ebiom.2024.104999

**Published:** 2024-02-09

**Authors:** Malin Barman, Monica Gio-Batta, Léna Andrieux, Mia Stråvik, Robert Saalman, Rikard Fristedt, Hardis Rabe, Anna Sandin, Agnes E. Wold, Ann-Sofie Sandberg

**Affiliations:** a Department of Life Sciences, Food and Nutrition Science, Chalmers University of Technology, Gothenburg 412 96, Sweden; b Institute of Biomedicine, Department of Infectious Diseases, Sahlgrenska Academy, University of Gothenburg, Gothenburg 405 30, Sweden; c Département de Biologie, École Normale Supérieure de Lyon, Université Claude Bernard Lyon 1, 69342 Lyon Cedex 07, France; d Institute of Clinical Sciences, Department of Pediatrics, University of Gothenburg, Gothenburg 405 30, Sweden; e Department of Clinical Science, Pediatrics, Sunderby Research Unit, Umeå University, Umeå 901 87, Sweden

**Keywords:** Short-chain fatty acids, Food allergy, Atopic eczema, Sensitisation, Breast milk, Infant/maternal plasma

## Abstract

**Background:**

Short-chain fatty acids (SCFAs) in intestinal contents may influence immune function, while less is known about SCFAs in blood plasma. The aims were to investigate the relation between infants’ and maternal plasma SCFAs, as well as SCFAs in mother’s milk, and relate SCFA concentrations in infant plasma to subsequent sensitisation and atopic disease.

**Methods:**

Infant plasma (N = 148) and corresponding mother’s milk and plasma were collected four months postpartum. Nine SCFA (formic, acetic, propionic, isobutyric, butyric, succinic, valeric, isovaleric, and caproic acid) were analysed by UPLC-MS. At 12 months of age, atopic disease was diagnosed by a pediatric allergologist, and sensitisation was measured by skin prick test. All families participated in the Swedish birth cohort NICE (Nutritional impact on Immunological maturation during Childhood in relation to the Environment).

**Findings:**

Infants with sensitisation, atopic eczema, or food allergy had significantly lower concentrations of five, three, and two SCFAs, respectively, in plasma at four months. Logistic regressions models showed significant negative associations between formic, succinic, and caproic acid and sensitisation [OR_adj_ (95% CI) per SD: 0.41 (0.19–0.91); 0.19 (0.05–0.75); 0.25 (0.09–0.66)], and between acetic acid and atopic eczema [0.42 (0.18–0.95)], after adjusting for maternal allergy. Infants’ and maternal plasma SCFA concentrations correlated strongly, while milk SCFA concentrations were unrelated to both. Butyric and caproic acid concentrations were enriched around 100-fold, and iso-butyric and valeric acid around 3-5-fold in mother’s milk, while other SCFAs were less prevalent in milk than in plasma.

**Interpretation:**

Butyric and caproic acid might be actively transported into breast milk to meet the needs of the infant, although mechanistic studies are needed to confirm this. The negative associations between certain SCFAs on sensitisation and atopic disease adds to prior evidence regarding their immunoregulatory potential.

**Funding:**

Swedish Research Council (Nr. 2013-3145 and 2019-0137 to A-S.S.), Swedish Research Council for Health, Working Life and Welfare FORTE, Nr 2018-00485 to A.W.), The Swedish Asthma and Allergy Association's Research Fund (2020-0020 to A.S.).

**Research in context**

**Evidence before this study**

Short-chain fatty acids (SCFAs) are produced by bacterial fermentation and are present in abundance in the large intestine. Small amounts are taken up across the gut wall and found in the circulation, but their low concentrations (1000-fold lower than in faeces) have precluded analysis using conventional methods, and their function needs to be better known. Breast milk also contains SCFAs; cow’s milk is rich in butyric and isobutyric acid. SCFAs can modulate immune functions, and low concentrations of certain SCFAs in faeces correlate with subsequent allergy development. To the best of our knowledge, concentrations of SCFAs in infant blood have not been investigated in relation to allergy development.

**Added value of this study**

SCFAs have been analysed in infant blood plasma in parallel with their mothers’ plasma and milk in >100 infant–mother pairs. We found much higher concentrations of butyric and caproic acid (around 100-fold) in breast milk compared to maternal plasma, while valeric and isobutyric acid were enriched 3 to 5-fold in mother’s milk. Other SCFAs were rather less prevalent in breast milk than in plasma. SCFA concentrations in infants’ plasma correlated strongly with SCFAs in maternal plasma but weakly with SCFAs in breast milk. Infants who were either sensitised or had atopic disease at 12 months of age had lower concentrations of some SCFAs in their plasma at four months of age than non-sensitised and non-allergic infants, adding to the evidence of SCFA as potential immune modulators, possibly contributing to healthy tolerance development.

**Implications of all the available evidence**

The higher concentrations of butyric and caproic acid in human milk call for further studies regarding the mechanism (selective transport or local production) and purpose (e.g., fuel for the infant’s gastrointestinal epithelium). Our study provides added evidence suggesting that SCFAs may modulate the immune system and be involved in physiologic tolerance development. The reason for the strong correlations between infant and maternal plasma concentrations of individual SCFAs should be studied further.

## Introduction

The prevalence of allergic diseases has increased dramatically over the last century in industrialised countries.^1^^,^^2^ Abundant microbial exposure via infections and/or commensal colonisation may provide the immune system with crucial maturation signals during a critical stage of development in infancy, necessary for the ability to actively tolerise harmless environmental antigens (“allergens”).^3^ The microbiota is established during the first years of life and becomes successively more complex over time.^4^ The low complexity of the gut microbiota during infancy and/or delayed maturation has been linked to an increased risk of subsequent atopic eczema,^5^^,^^6^ asthma,^7^ and food allergy.^8^

A group of metabolites that could link early gut colonisation with immune maturation are the short-chain fatty acids (SCFAs) that are produced when anaerobic bacteria ferment carbohydrates from the diet, colonic mucus and sloughed epithelial cells.^9^ SCFAs are carboxylic acids with one to six carbon atoms, including formic (1 C), acetic (2 C), propionic (3 C), butyric (4 C), valeric (5 C) and caproic acid (6 C). In addition, branched varieties, such as isobutyric and isovaleric acids, are chiefly derived from the fermentation of amino acids.^10^^,^^11^ Succinic acid (4 C), which has double carboxyl groups, is also produced by gut microbes and may be grouped with the SCFAs. The faecal SCFA pattern changes alongside the maturation of the infant’s microbiota. Thus, while formic and acetic acid are produced by the microbiota of newborn infants, longer and branched SCFAs are not produced in significant amounts until the microbiota has become more complex and dominated by obligate anaerobes.^12^ In addition to the production of SCFAs by colonic bacteria, these fatty acids can be consumed directly via the diet (e.g., butyric acid in dairy products).^13^, ^14^, ^15^

SCFAs are absorbed by the gut epithelium via diffusion, carrier-mediated transport, and exchange with bicarbonate.^16^ SCFAs serve as fuel for colonic mucosal epithelial cells, with butyric acid as the preferred energy substrate.^17^ SCFAs promote epithelial barrier integrity and stimulate mucus production.^18^, ^19^, ^20^ They may also influence a range of immune functions.^18^^,^^21^, ^22^, ^23^ Several studies have demonstrated a negative correlation between high concentrations of certain SCFAs in infant faeces and subsequent allergy development.^12^^,^^24^, ^25^, ^26^ In a recent cross-sectional study, lower breast milk concentrations of acetic acid were also negatively associated with atopic dermatitis severity in infants aged 2–4 months.^27^

Varying amounts of the different SCFAs pass into the portal vein^28^ and reach the systemic circulation,^29^^,^^30^ where they are involved in various physiologic processes, including hepatic gluconeogenesis (propionic acid), *de novo* lipogenesis (acetic acid)^29^^,^^31^ and blood pressure regulation (valeric acid).^30^ When 13C-labelled SCFAs were introduced in the colon, 36% of the acetic acid, 9% of the propionic acid, and 2% of the butyric acid reached the circulation.^29^ Regarding SCFAs in breast milk, studies have identified formic, acetic, butyric, and succinic acids as the most abundant.^32^, ^33^, ^34^, ^35^ However, the quantitative relation between plasma and breast milk SCFA concentrations has been little studied. Breast milk SCFAs could provide a source for circulating SCFAs, but to the best of our knowledge, this has not been studied in humans.

Endogenous production is a third source of circulating SCFA besides diet and colonic fermentation. Formic acid may derive from the catabolism of serine, glycine, methionine, and choline.^36^^,^^37^ Acetic acid is the major metabolite of ethanol.^38^ Succinic acid is a critical intermediate in the citric acid cycle,^39^ with blood concentrations increased after physical exercise,^40^ and acetic acid is also released into the circulation and taken up again through acetyl-CoA hydrolase and synthetase.

SCFA concentrations are 1000 times lower in plasma than in faeces,^28^^,^^41^ and while SCFAs in faeces have been investigated since the 1970s, the low concentrations of SCFAs in plasma have only recently been adequately measured using highly sensitive techniques, such as ultraperformance liquid chromatography-mass spectrometry (UPLC-MS).^42^ SCFA concentrations in plasma have been related to specific metabolic markers,^41^ asthma,^43^ and obesity^44^ in adults. However, to our knowledge, concentrations of circulating SCFAs in early life have not yet been investigated in relation to allergy development.

The aim of this work was to measure SCFA levels in infant plasma and relate these levels to SCFAs in corresponding maternal plasma and mother's milk and to investigate if plasma SCFA levels in infancy were related to subsequent sensitisation and atopic disease. We employed a highly sensitive methodology to measure nine different SCFAs in blood plasma obtained from ∼140 four-month-old infants together with corresponding maternal plasma and breast milk.

## Methods

### Study population

A total of 148 infant–mother pairs were selected from the NICE (Nutritional impact on Immunological maturation during Childhood in relation to the Environment) birth cohort comprising 651 children and their parents, ClinicalTrials.gov ID: NCT05809479. Expecting parents with planned delivery at Sunderby Hospital in Northern Sweden were recruited in 2015–2018. The families were followed with repeated questionnaires and collection of biological samples until the child reached six years of age.^45^ The inability to communicate in Swedish was the only exclusion criterion. Approximately 10% of all deliveries in the catchment area were successfully recruited to the NICE cohort.^46^ More detailed information about the whole cohort has previously been published in the study protocol.^45^

### Sampling of mothers and infants at four months postpartum

Venous blood from both mothers and infants was collected in EDTA tubes at a study visit four months postpartum. The plasma was separated and stored frozen at −80 °C until analysed.^45^ Breast milk was collected at home before the study visit, kept in the freezer, and brought frozen to the clinic. The mothers were instructed to collect 30 mL of breast milk (by hand or with a pump) just before breastfeeding the baby during the first part of the day (i.e., before noon).

### Assessment of mothers’ dietary intake at four months postpartum

Maternal food intake was assessed using a web-based, validated, semi-quantitative food frequency questionnaire (Meal-Q) sent out four months postpartum.^47^^,^^48^ The questionnaire reflected food intake during the past month (i.e., between three and four months postpartum). Intake in grams per day of a wide range of food items was estimated using reported intake frequency and estimated portion sizes based on pictures shown to the interviewees.

### Assessment of atopic disease and sensitisation to common allergens

At 12 months of age, the infants underwent clinical examination by the study paediatrician (author AS). The examination included careful clinical history and evaluation of symptoms. Diagnosis of atopic disease included in the statistical calculations in this paper was based on the criteria listed in Table 1. Skin prick test to a panel of common allergens was performed using standardised allergens (Soluoprick, ALK Nordic A/S, Denmark) according to a standardised procedure at 12 months of age^49^ and evaluated as specified in Table 1. In conjunction with this visit, the parents were asked about their own (doctor’s diagnosed) atopic conditions using a structured questionnaire.

**Table 1:** Criteria for diagnosis of atopic diseases and sensitisation.

	Criteria for diagnosis
Sensitisation	A wheal diameter of ≥3 mm after 15 min to at least one of six common allergens (hen’s egg, cow's milk, birch, timothy, cat, and dog) in skin prick test.
Atopic eczema	Major criterion + ≥3 minor criteria fulfilled ***Major criterion:*** An itchy condition, usually manifested as scratching/rubbing. ***Minor criteria:*** (1) itchiness or visible eczema in typical areas such as cheeks, folds of the elbows or knees, or front of ankles or around the neck; (2) general dryness of the skin; (3) asthma, hay-fever, or another atopic disease; (4) a history of atopic disease in a first-degree relative.
Food allergy	Typical symptoms appearing in relation to the intake of a triggering food(s) confirmed by ≥ 1 oral food challenge in which symptoms disappeared as the suspected offending food was eliminated from the infant’s diet, and the same symptoms reappeared upon challenge.

Sensitisation was assessed by skin prick testing at one year of age.

Atopic disease was diagnosed by a specialist in pediatric allergology at a clinical visit at one year of age.

### Selection of samples for SCFA analysis

As the aim of the study was twofold: 1) to study the relation between SCFA in infant plasma and corresponding mother’s milk and plasma and 2) to investigate if low plasma SCFA in infancy was a risk factor for allergic diseases, we selected the following samples for SCFA analysis: 1) all complete sets of four months samples (infant plasma, maternal plasma, and breast milk), regardless of whether the child later developed atopic disease or became sensitised or not, 2) all atopic children that had not been included under 1), provided there was a plasma sample obtained at four months, regardless of the availability of maternal plasma or breast milk (Table 2).

**Table 2:** Included infants and analysed samples.

Allergy diagnosis at 12 months	Number of samples		
	Infant plasma	Maternal plasma	Mother’s milk
Non-atopic^a^	109	105	107
Any atopic disease or sensitisation	28	27	13
Atopic eczema	19	19	8
Food allergy	14	13	7
Sensitised	11	11	6
Others^b^	11	10	8
Total	148	142	128

a Non-allergic and non-sensitised.

b Infants with other atopic conditions (e.g., non-atopic asthma or conjunctivitis) or with an uncertain diagnosis of e.g., food allergy.

### Analysis of short-chain fatty acids in plasma and breast milk

Eight SCFAs (formic, acetic, propionic, isobutyric, butyric, isovaleric, valeric, and caproic acid) and the di-acid succinic acid (hereafter collectively termed SFCAs) were analysed by UPLC-MS as described previously,^42^ with modifications.^50^ Ten μl plasma was incubated with 60 μl 75% methanol (LiChrosolv®), 10 μl 200 mM 3-NPH, and 10 μl 120 mM EDC-6% pyridine at room temperature for 45 min with shaking. The reaction was quenched with 10 μl of 200 mM quinic acid during 15 min shaking. After centrifugation (15,000 g, 5 min), the supernatant was collected, and 10% methanol in water was added up to 1 mL, centrifuged anew, and 100 μl derivatised sample was mixed with 100 μl of internal standard (^13^C6-3NPH, IsoSciences Inc., King of Prussia, PA, USA). The same 13C standard was used for plasma and breast milk and showed the same area, indicating that there is no matrix effect induced by the breast milk matrix compared to plasma. A 6500+ QTRAP triple-quadrupole mass spectrometer (AB Sciex, 11,432 Stockholm, Sweden) equipped with an APCI source and operated in the negative-ion MRM-mode was used for detection after separation on a Waters ACQUITY UPLC BEH C18 column, 1.7 μm 2.1 × 150 mm at 0.4 mL/min flow rate, 40 °C column temperature, the autosampler kept at 5 °C. For the gradient, 0.5% solvent B (LCMS grade acetonitrile) in solvent A (LC-MS grade water) was held for 3 min, followed by 2.5% solvent B for 3 min, ramping linearly to 17% at 6 min, 45% at 10 min and 55% at 13 min, followed by a flush (100% solvent B) and recondition (0.5% solvent B), in a total run time of 15 min. Three different QC plasma samples and three different QC breast milk samples were used in triplicates in each batch to follow batch effects.

The scheduled MRM transitions were optimised by direct infusion of the derivatives containing ten μM of each fatty acid. The Q1/Q3 pairs were used in the MRM scan mode to optimise the collision energies for each analyte, and the two most sensitive pairs per analyte were used for subsequent analyses. All compounds were purchased from Sigma–Aldrich (Solkraftsvägen 14C, 135 70 Stockholm).

### Ethics

The study was conducted in accordance with the Helsinki Declaration and approved by the Regional Ethical Review Board in Umeå, Sweden (2013/18-31M, 2015-71-32); all parents signed written consents for themselves and their children.

### Statistics

Non-parametric statistical approaches were chosen due to data not being normally distributed. More specifically, Mann–Whitney U test was used to compare SCFA concentrations between the different diagnostic groups. Logistic regression was used to examine the effect sizes of the associations and to adjust associations between SCFA concentrations and infant atopic diseases for maternal atopy. In these models, SCFA concentrations were converted to standardised variables and used as continuous exposure variables in separate models, with infant atopic disease as a binary outcome variable (non-atopic = 0, atopic = 1) with non-atopic being the reference, and maternal atopy (non-atopic = 0, atopic = 1) as a binary covariate. Due to the sample size, no other covariates were included in the analyses.

Spearman rank correlations were used to assess associations between diet and SCFA concentrations in breast milk and plasma, and correlation size was interpreted based on Cohen’s criteria.^51^ For comparing background data between any two groups, Fisher’s exact test or Pearson Chi-Square was used for categorical variables, and the Mann–Whitney U test was used for continuous variables. p-values (two-tailed) below 0.05 were considered significant for all tests. Data were analysed using IBM SPSS version 28 (IBM, New York, NY, USA), GraphPad Prism version 9 (GraphPad, San Diego, USA), and R version 4.1.2 (R Foundation for Statistical Computing, Vienna, Austria).

### Role of funders

This research was funded by the Swedish Research Council, grant number 521-2013-3154 and grant number 2019-0137, Swedish Research Council for Health, Working Life and Welfare (FORTE) grant number 2018-00485, and the Innovation Unit at Region Norrbotten. The funders were not involved in the study design, collection, analysis, interpretation of data, the writing of this article, or the decision to submit it for publication.

## Results

Nine SCFAs ranging from 1 to 6 carbon atom chain length were analysed in 148 plasma samples from infants, 142 plasma samples from mothers, and 128 breast milk samples, all collected four months postpartum. Among the N = 128 individuals with donated breast milk samples, N = 126 had responded to questionnaires regarding extent of breastfeeding at the time of sampling. Out of these, N = 104 (83%) were exclusively breastfed and N = 22 (17%) was partially breastfed at that time.

**Fig. 1:**
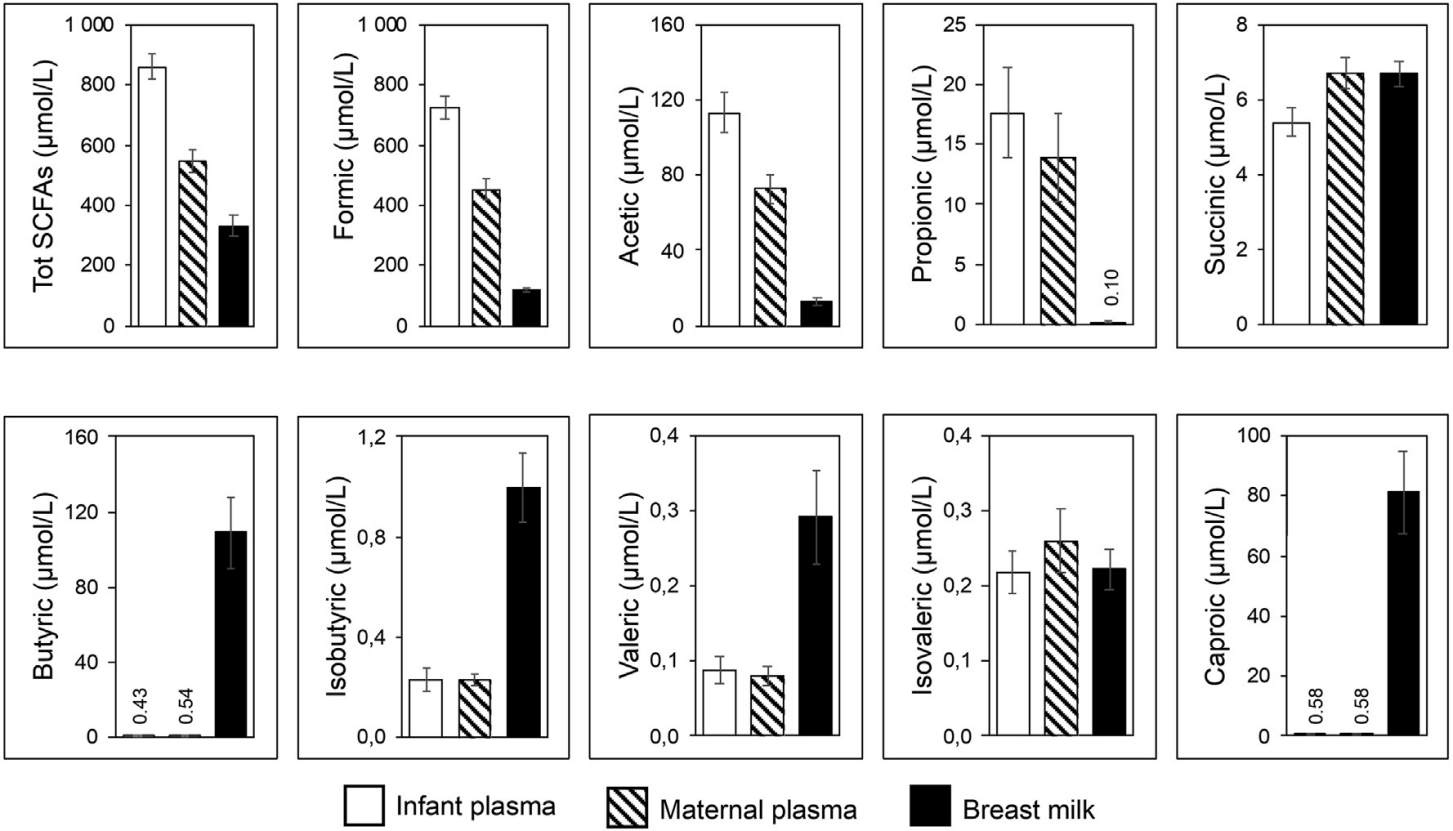
Median concentrations (μmol/L) of short-chain fatty acid (SCFA) concentrations in infant plasma (white bars, N = 148), maternal plasma (striped bars, N = 142), and breast milk (black bars, N = 128). Error bars, 95% CI.

The total SCFA concentrations were higher in infants’ plasma than in mothers’ plasma (850 vs. 550 μmo/l, p < 0.001), but lower in breast milk than in maternal plasma (250 vs. 550 μmol/L, p < 0.001) (Fig. 1, Supplemental Table S1).

Formic acid was the most abundant SCFA in both infant and maternal blood plasma (84% of the total SCFA concentration for both), followed by acetic acid (11% for both) and propionic acid (1% for both) (Supplemental Table S1). Butyric acid and the longer and branched SCFAs (i.e., isobutyric acid, valeric acid, isovaleric acid and caproic acid) were found in very low concentrations in plasma <1 μmol/L (Fig. 1). Compared to their mothers, infants had higher concentrations of the shortest SCFAs (formic acid, acetic acid, and propionic acid) in their plasma, but equal or somewhat lower concentrations of the longer or branched SCFAs (Fig. 1).

While most SCFAs were found in lower concentrations in breast milk than in plasma, butyric and caproic acid were found at 100 and 90 times, respectively, higher concentrations in breast milk as compared to maternal plasma. Isobutyric acid and valeric acid concentrations were also higher in breast milk as compared to plasma. Thus, butyric acid made up 25% and caproic acid 20% of the breast milk SCFAs, while they made up <1% of the plasma SCFAs (Fig. 1, Table 3).

**Table 3:** Ratio of concentrations of various short-chain fatty acids (SCFAs) in breast milk vs. maternal plasma.

SCFA	No. of carbon atoms	Ratio breast milk/maternal plasma concentration Median (25th–75th percentile)
Formic acid	1	0.32 (0.20–0.46)
Acetic acid	2	0.18 (0.089–0.29)
Propionic acid	3	0.0044 (0.000–0.050)
Butyric acid	4	100 (46–270)
Isobutyric acid	4	4.6 (2.3–7.2)
Succinic acid	4	1.0 (0.72–1.3)
Valeric acid	5	2.9 (0.67–8.9)
Isovaleric acid	5	1.1 (0.43–3.4)
Caproic acid	6	89 (48–200)
Total SCFAs	–	0.64 (0.36–0.92)

Total SCFAs = sum of the nine measured short-chain fatty acids. Paired breast milk and blood plasma samples of 125 mothers were analysed by UPLC-MS. Samples were obtained four months postpartum.

Ratios between infant and maternal plasma and ratios between infant plasma and breast milk are found in Supplemental Table S1.

### Correlation between SCFA concentration in infant and maternal plasma, and mother’s milk

**Fig. 2:**
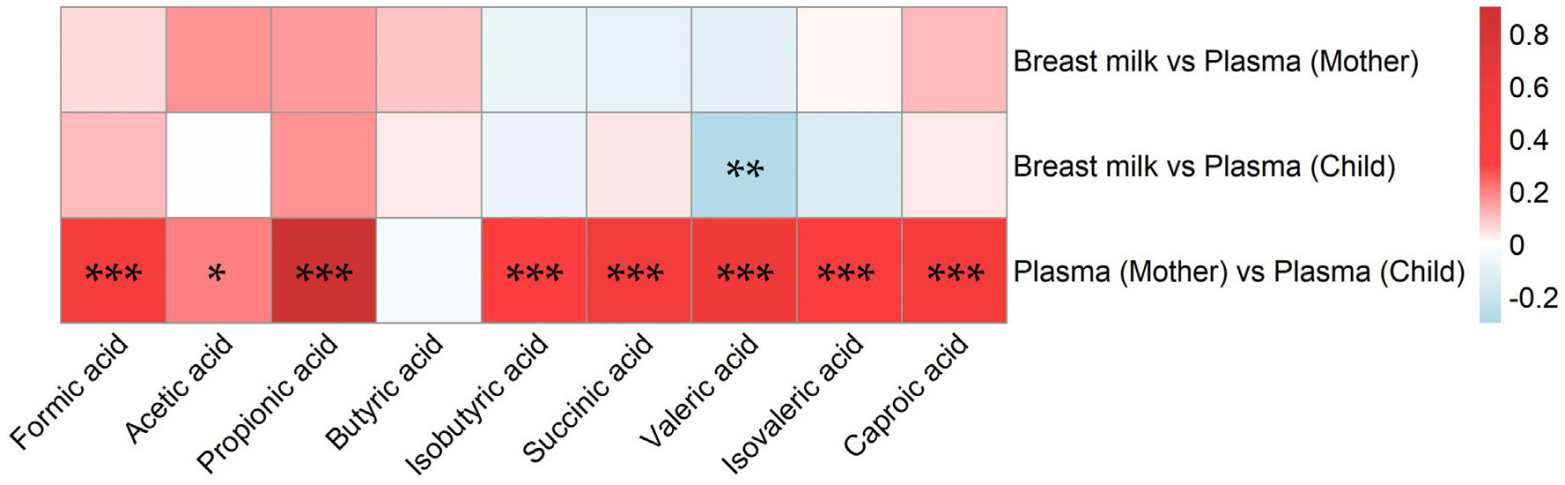
Correlations between maternal plasma and breast milk and infant plasma concentrations of individual SCFAs (Spearman’s rank correlation). Red color denotes a positive correlation; blue color denotes a negative correlation. Number of samples: breast milk vs. maternal plasma, N = 125; breast milk vs. infant plasma, N = 128; maternal plasma vs. infant plasma, N = 142. ∗p < 0.05, ∗∗p < 0.01, ∗∗∗p < 0.001. Rho and p-values are specified in Supplemental Table S2.

We first studied the correlation between individual SCFAs in the mother’s plasma and her breast milk (Fig. 2, uppermost lane). There were no significant correlations between any of the SCFAs in the mother’s plasma and her breast milk.

Next, we correlated the concentrations of individual SCFAs in mother’s milk and infants plasma. No significant positive correlations were noted; instead, valeric acid in mother's milk and infant plasma correlated negatively (rho = −0.27, p = 0.002) (Fig. 2, middle lane). In secondary analyses, with only exclusively breastfed infants (N = 104), the results regarding valeric acid remained (rho = −0.32, p = 0.001). In addition, formic acid and propionic acid in mother's milk now correlated significantly with the concentrations in infant plasma (rho = 0.22, p = 0.024, and rho = 0.24, p = 0.013, respectively). SCFA concentrations in maternal plasma and mother's milk did not correlate with each other (Fig. 2, upper lane).

Lastly, we correlated the infants’ plasma concentrations of SCFA with their mothers’ plasma concentrations. Maternal and infant plasma concentrations correlated significantly, with butyric acid as the single exception (Fig. 2, bottom lane). The strongest correlations were observed for propionic (rho = 0.89), valeric (rho = 0.56) and succinic acid (rho = 0.51) (p < 0.001 for all). Caproic, formic, isovaleric, and isobutyric acid all correlated moderately (rho = 0.36–0.49, p < 0.001), and acetic acid concentrations correlated weakly (rho = 0.20, p = 0.017). Secondary analyses were done on the seven non-breastfed infants since their plasma concentrations could not have been affected by intake of mother’s milk. Strong correlations remained for propionic acid (rho = 0.79, p = 0.036) and succinic acid (rho = 0.89, p = 0.007), as did the moderate correlations seen for formic, valeric, and caproic acid (rho = 0.32–0.36), although the latter lost statistical significance, not surprisingly considering the low number of observations (n = 7). This indicated that associations between maternal and infant plasma were not driven by the infant’s consumption of breast milk.

### Mothers’ diet and SCFA concentrations in maternal plasma and breast milk

**Fig. 3:**
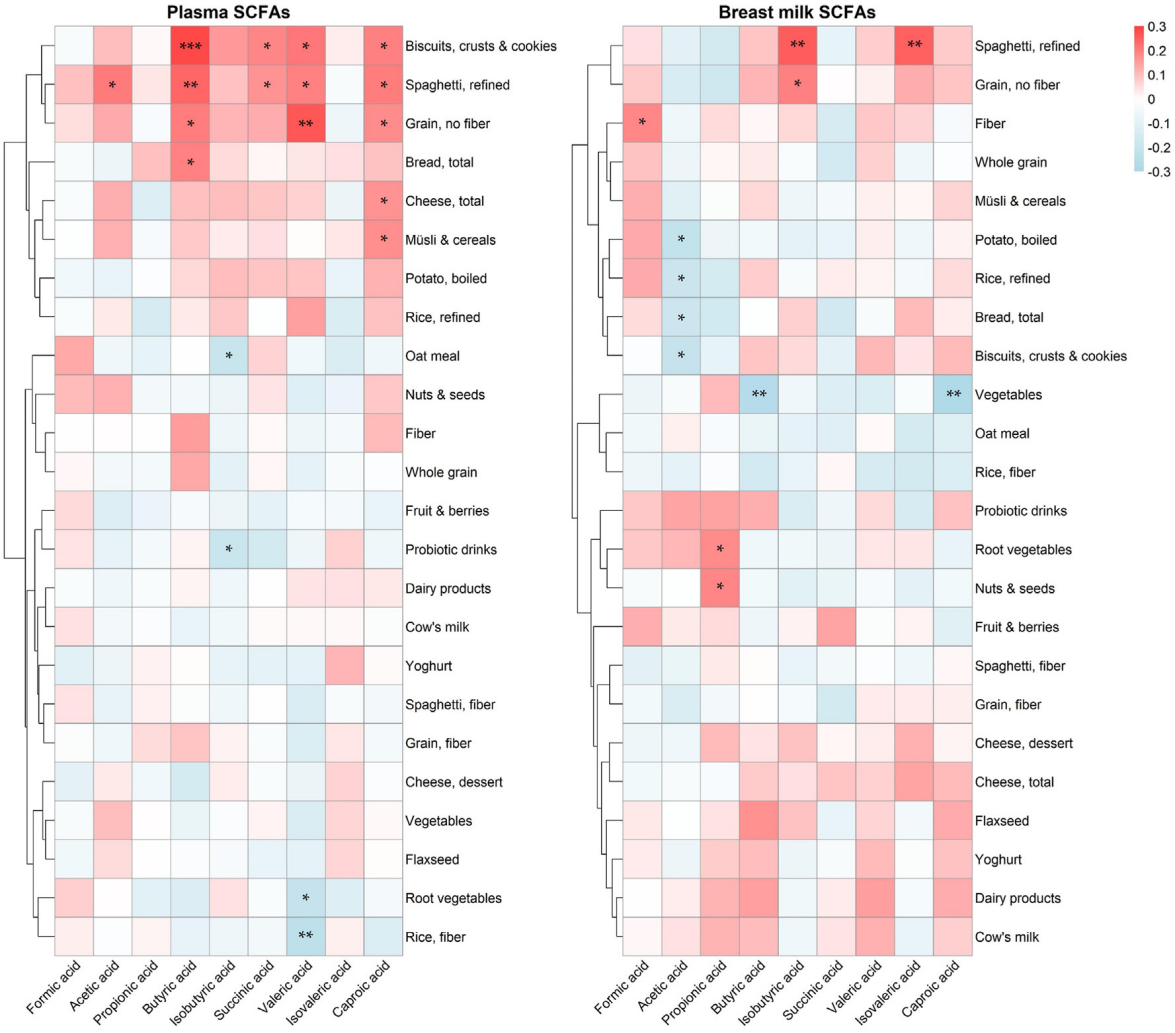
Spearman correlations between dietary intake and SCFAs in maternal plasma and breast milk. Food intake was assessed to reflect maternal intake between 3 and 4 months postpartum. Significant correlations are denoted with asterisks as p < 0.001 = ∗∗∗, p < 0.01 = ∗∗, and p < 0.05 = ∗. The red color indicates a positive correlation. The blue color denotes a negative correlation.

The mother’s food intake was assessed by FFQ combined with the estimated portion size at 4 months postpartum, covering the last month (3–4 months postpartum). The association between intake of different food items and the concentrations of eight SCFAs in plasma and breast milk were investigated by correlation analysis (Fig. 3). SCFA concentrations did not correlate with the reported intake of fiber-rich foods or whole grain products, except for a weak correlation between fiber intake (based on nutritional calculations from the whole diet) and concentrations of the shortest SCFA, formic acid, in breast milk (rho = 0.19, p = 0.037), and between intake of nuts and seeds and propionic acid in breast milk (rho = 0.19, p = 0.035). Instead, consumption of refined varieties of spaghetti and grains was positively associated with several SCFAs. For instance, the reported intake of biscuits, crusts and cookies correlated positively with butyric acid in plasma, and the reported intake of refined spaghetti correlated positively with breast milk concentrations of isobutyrate and isovalerate (Fig. 3).

### SCFAs in infant plasma at four months vs. sensitisation and atopic disease at 12 months

**Fig. 4:**
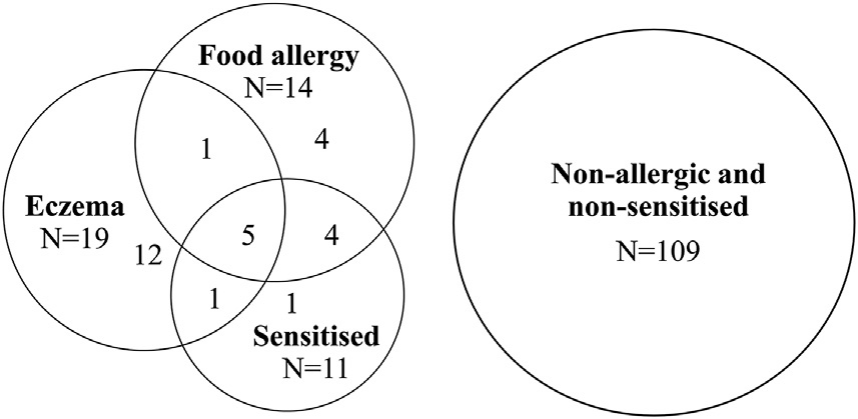
Distribution of atopic conditions of the infants who were included in the statistical calculations.

In total, 28 infants had either one or more atopic diseases or were sensitised at 12 months of age (Fig. 4). The SCFA concentrations at four months of age were compared between infants who had either atopic eczema and/or food allergy and/or were sensitised, and the 109 infants who were neither sensitised nor obtained any allergy diagnosis by 12 months. Infants with other atopic conditions (e.g., non-atopic asthma or conjunctivitis) or with uncertain diagnosis, N = 11, were not included in the statistical analyses regarding allergy and sensitisation.

**Fig. 5:**
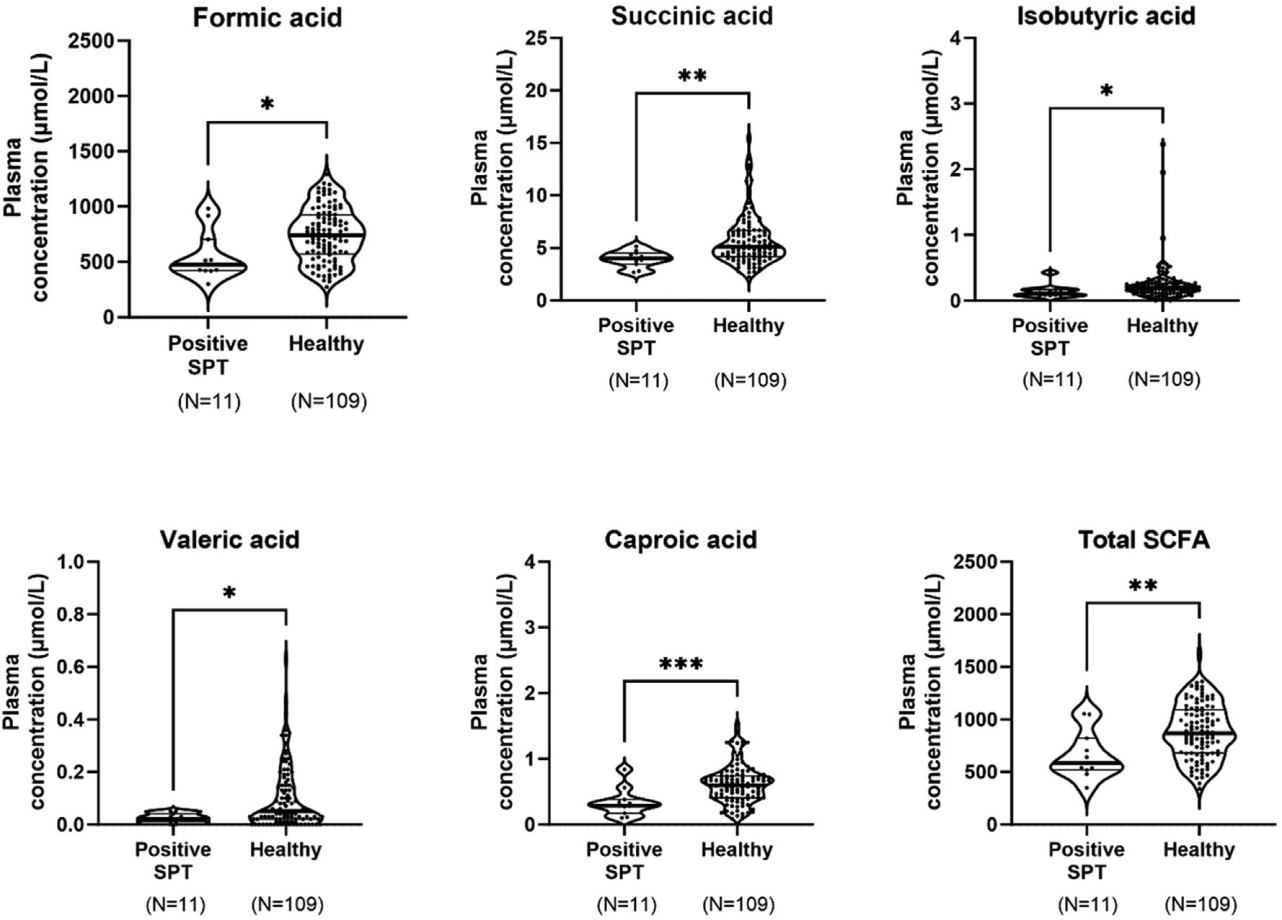
Difference in SCFA concentrations in plasma at four months of age in those who were sensitised at 12 months of age (N = 11) compared to infants who were non-allergic and non-sensitised at 12 months of age (N = 109). Allergic sensitisation was defined as a positive skin prick test (>3 mm) to at least one allergen. The following SCFA concentrations did not differ significantly between the groups and are therefore not displayed: acetic acid, propionic acid, butyric acid, and isovaleric acid. Differences in SCFA concentrations were measured with Mann–Whitney U-test.

Infants who were sensitised at 12 months had lower concentrations of several SCFAs at four months of age than the non-atopic, non-sensitised children, including formic (p = 0.01), succinic (p = 0.002), isobutyric (p = 0.04), valeric (p = 0.03), and caproic acid (p < 0.001) as well as a lower concentration of total SCFAs (p = 0.008) (Fig. 5).

**Fig. 6:**
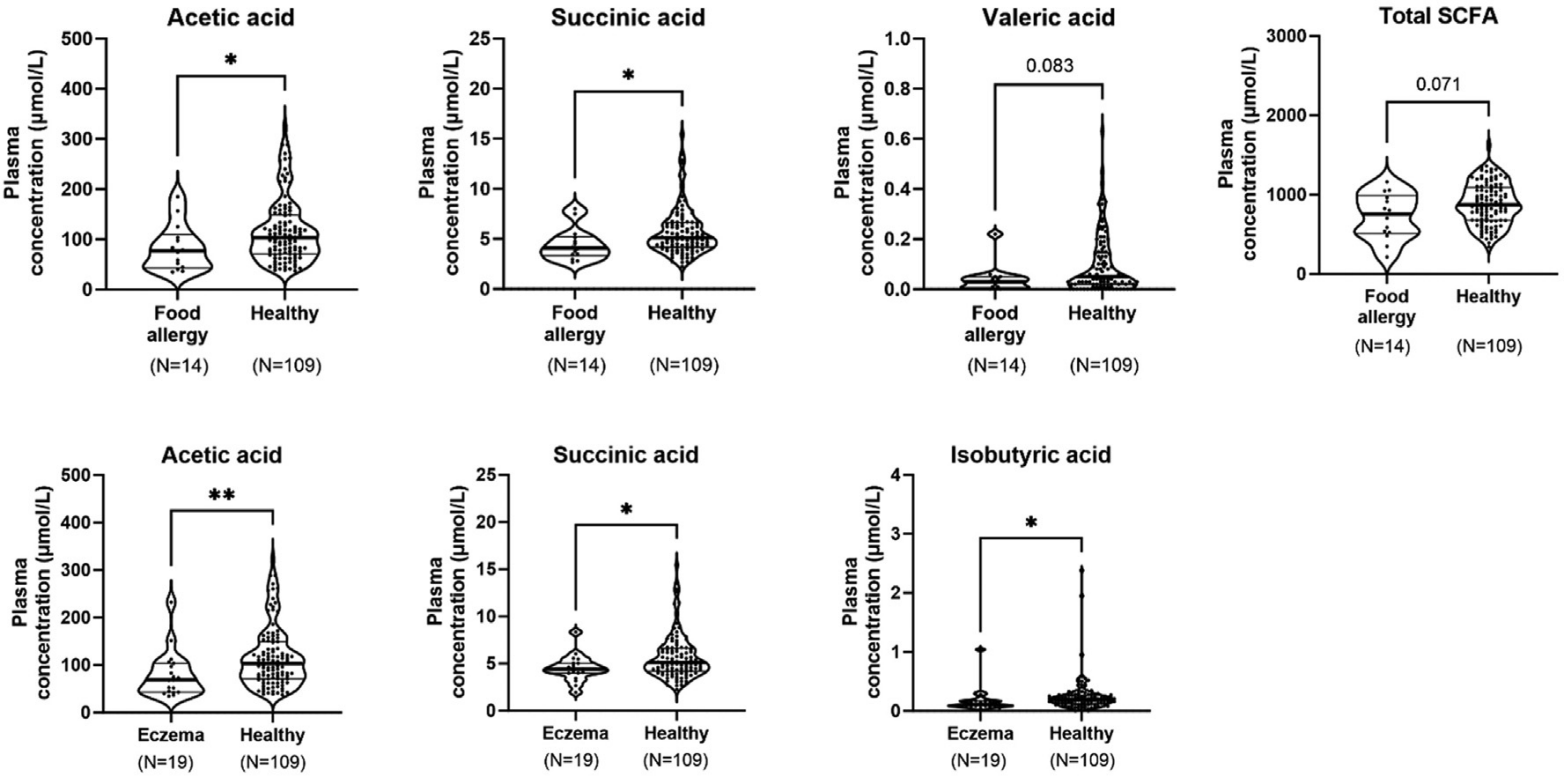
Difference in SCFA concentrations in infant plasma between infants with food allergies (N = 14, upper part) or atopic eczema (N = 19, lower part) and non-allergic, non-sensitised infants (N = 109). The following SCFA concentrations did not differ significantly between the groups and are therefore not displayed: formic acid, propionic acid, butyric acid, isovaleric acid, and caproic acid. Differences in SCFA concentrations were measured with Mann–Whitney U-test.

Further, infants diagnosed with food allergy at 12 months of age had lower concentrations of acetic acid (p = 0.04) and succinic acid (p = 0.03) in their plasma at four months of age compared to non-allergic, non-sensitised infants (Fig. 6). They also tended to have lower concentrations of valeric acid (p = 0.08) and total SCFAs (p = 0.07). Infants with atopic eczema had lower concentrations of acetic acid (p = 0.003), succinic acid (p = 0.03), and isobutyric acid (p = 0.04) than non-atopic infants (Fig. 6).

### SCFA concentrations in breast milk in relation to sensitisation and infant allergy

No differences in SCFA concentrations in mother’s milk at four months of age and atopic disease and/or allergic sensitisation at twelve months of age were observed.

### Mothers’ atopic diseases and SCFA concentrations in maternal plasma and breast milk

Mothers and fathers were asked about their own doctors’ diagnosed allergic conditions in conjunction with the clinical evaluation of the children at 12 months. SCFAs in maternal plasma and breast milk at four months postpartum were related to her allergic conditions. Allergic mothers had lower concentrations of several SCFAs compared to non-allergic mothers, most consistently seen for isobutyric and succinic acid, while isovaleric acid was generally higher in the plasma of allergic mothers, as compared to non-allergic mothers. Breast milk concentrations of SCFA were mostly unrelated to the atopic state of the mother, except for a lower concentration of caproic acid and a tendency toward lower butyric acid concentrations in allergic mothers.

### Effect sizes of associations between SCFAs in infants' plasma and subsequent sensitisation and atopic disease with and without adjustment for maternal allergy

Allergy is partly inherited and here, N = 57 (39%) of the mothers had any allergic disease (compared to N = 91 (61%) without any allergic disease). Indeed, any type of maternal allergy was significantly more prevalent among children with atopic eczema (58% as compared to 34% among non-atopic, non-sensitised children) but also more prevalent, although non-significantly, among children with food allergy (57%) and among sensitised children (55%) (Supplemental Table S3). In addition, maternal allergy to pets was significantly associated with atopic eczema in the child, and maternal asthma was significantly associated with food allergy in the child (Supplemental Table S3).

To investigate the effect sizes of the associations between SCFAs in infants’ plasma and subsequent sensitisation and atopic disease and to investigate whether the associations were mediated via maternal allergy, logistic regression models were performed (Supplemental Tables S4–S6). The logistic regression models resulted in fewer significant associations compared to the previously shown analyses of median concentrations in plasma between infants with and without atopic diseases/sensitisation. Higher concentrations of formic, succinic, and caproic acid in plasma at 4 months were significantly associated with lower odds of being sensitised at 12 months of age [OR (95% CI) per SD: 0.39 (0.18–0.87); 0.17 (0.04–0.68); 0.23 (0.09–0.62), respectively]. The associations remained after adjusting for maternal allergy: [OR_adj_ (95%CI) per SD: 0.41 (0.19–0.91); 0.19 (0.05–0.75); 0.25 (0.09–0.66)]. For atopic eczema, high levels of acetic and succinic acid were associated with lower odds in the unadjusted models [OR (95% CI) per SD: 0.39 (0.17–0.88) and 0.44 (0.20–0.96), respectively]. However, only the association between succinic acid and atopic eczema remained after adjusting for maternal allergy [OR_adj_ (95% CI) per SD: 0.48 (0.22–1.05)]. SCFAs in infant plasma were not associated with odds for food allergy, contrary to the results found when comparing means of concentrations with the Mann–Whitney U-test.

## Discussion

This study aimed to measure SCFAs in plasma of infants and their mothers, as well as in mother’s milk, and to investigate these concentrations in relation to sensitisation and allergic disease during the first year of life. In here, we measured concentrations of nine SCFAs (formic, acetic, propionic, isobutyric, butyric, isovaleric, valeric, succinic and caproic acid) in the blood plasma of infants at four months of age and in their mothers’ blood plasma and breast milk obtained at the same time point.

Our sensitive analytical method enabled us to measure several SCFAs that have not previously been reported to be present in breast milk, including caproic, isobutyric, propionic, and valeric acid. A notable finding was that butyric and caproic acid were both enriched around 100-fold in breast milk as compared to the blood plasma of the lactating woman. Valeric and isobutyric acid were also enriched, although a mere 3-5-fold. Other SCFAs were 4–10 times less abundant in breast milk as compared to blood plasma, as was the total SCFA concentration. Several studies have reported the presence of formic, acetic, and butyric acid in human breast milk in concentrations comparable to those found by us.^32^, ^33^, ^34^, ^35^ However, although a review pointed to the relatively higher concentrations of butyric acid in the milk as compared to serum,^52^ this conclusion was drawn from comparing studies that measured SCFA either in breast milk or blood plasma, not both fluids in the same study. Our findings prove that butyric and caproic acid are, indeed, enriched in the milk during lactation and raise questions regarding the mechanisms for this enrichment as well as its physiological purpose.

Mammary gland epithelium produces medium-chain fatty acids for export into the milk. In the cow, mammary epithelial cells can both produce butyric *de novo* and also use acetate and β-hydroxybutyrate (a metabolite of butyrate formed after its absorption from the rumen) absorbed from the blood as precursors for the synthesis of longer fatty acids.^14^^,^^53^ Humans and other non-ruminants instead synthesise medium size milk C8-14 and possibly also C16-18 fatty acids *de novo* using glucose as starting material^52^^,^^54^^,^^55^ and, to the best of our knowledge, *de novo* synthesis of butyrate or other SCFA has not been demonstrated in the non-ruminant mammary gland. More likely, the enrichment of butyric acid and caproic acid demonstrated here is achieved by transport from the plasma into human milk against a concentration gradient.

Several classes of SCFA transporters have been identified in colonic epithelial cells of which the proton-coupled monocarboxylate transporter MCT1 serves as the major transporter of butyric acid from the colonic lumen into colonic epithelial cells, and also out of the epithelial cell into the blood stream.^56^ MCT1 is widely distributed^57^ and is expressed intensely on the basolateral aspect of mammary gland epithelial cells, especially during lactation.^58^ However, MCT1 transports SCFA along a concentration gradient and not against it,^56^ making it an unlikely candidate for pumping butyric and caproic acid against a 100-fold concentration gradient. Another interesting candidate is BCRP (breast cancer resistance protein) that was initially discovered in multidrug resistant breast cancer cell lines from which it pumps chemotherapeutic drugs out of the cell.^59^ It was renamed ABCG2 when found to be a member of the ATP-binding cassette (ABC) transporter superfamily, that mediates active ATP-dependent transport against a concentration gradient. BCRP transports butyric acid into colonic epithelial cells^60^ Further, it is expressed in mammary alveolar epithelial cells during late pregnancy and lactation and pumps a variety of small compounds against a concentration gradient into the milk.^61^ Lastly, mutations in BCRP/ABCG2 have major effects on milk yield and fat composition of cow’s milk,^62^ indicating its involvement in mammary gland fat synthesis in the cow, which is dependent on butyrate. Further research is needed to establish the mechanism by which butyric and caproic acid, and to a lesser extent isobutyric and valeric acid, are enriched in human milk.

Butyric acid is a fuel for the colonic mucosa and acetic, propionic, and butyric acid also play important roles as substrates for the body’s glucose, cholesterol, and lipid metabolism.^63^^,^^64^ Accordingly, the butyric acid content of human milk was recently shown to influence infant body weight.^32^, ^33^, ^34^, ^35^ We therefore propose that the SCFAs in human milk are there to meet the needs of the infant, promoting e.g., epithelial integrity, metabolic balance and control of inflammation.^57^^,^^65^^,^^66^ Absorption of SCFA from the breast milk might take place in the upper gastro-intestinal tract, as the active butyric acid transporter BCRP, mentioned above, is more avidly expressed in the duodenum than in the colon.^67^ Breast milk SCFAs could, thus, chiefly serve to nourish the upper gastro-intestinal epithelia. Furthermore, breast milk would supply butyric and caproic acid at a very young age, when the microbiota has not achieved a sufficient complexity to produce these rather complex SCFAs which only appear in substantial quantities toward the end of the first year of life.^12^

When we compared the concentrations of individual SCFAs in mothers’ milk with those in their infants’ blood plasma, we found only weak correlations for formic and propionic acid. We surmise that most of the milk SCFA are either used by the epithelia or converted into glucose or longer fatty acids and/or glucose by the liver when arriving via the portal vein^63^^,^^64^ and therefore only to a very limited degree end up in the intact form in the circulation of the infant.

In contrast, we found surprisingly consistent and often strong correlations between infant and maternal concentrations of all SCFAs, with the exception of butyrate. We cannot easily explain this finding. It cannot result from transport from mother to infant during pregnancy^68^ since SCFA concentrations in plasma have a half-life of only a couple of hours,^29^ and we measured SCFA in maternal and infant plasma four months postpartum. Furthermore, it is highly unlikely that mothers and infants share the same SCFA pattern in the colon. Young infants lack most of the very obligate anaerobes and produce mostly the simplest SCFA, formic and acetic acid, in faeces at four months, while more complex SCFA are only produced when the infants are some years older.^12^ Mothers and infants also do not share diets since almost all of the infants in this cohort were breastfed (78% exclusively and 17% partially). To speculate, the significant correlations between infant and maternal SCFA concentrations could depend on endogenous production and/or tightly regulated uptake, and/or consumption, which could be under genetic control, and, thus, shared between parent and child. For instance, formic acid is a source molecule for one-carbon metabolism, and a whole-body loop exists for its endogenous production and regeneration.^36^^,^^37^ Also, acetic acid in the form of acetyl coenzyme-A is a fundamental molecule in cellular metabolism,^69^ and succinic acid is an intermediate in the TCA cycle.^39^ It would be interesting to study whether the fathers’ plasma SCFA patterns correlate equally strongly with their infants’, which would be the case if genetics had a strong influence. Interestingly, the contribution of host genetics to circulating SCFA levels was recently shown in two independent, healthy cohorts.^70^

We found no association between the reported intake of dietary fiber and SCFA concentrations in the plasma of the mothers, except for weak associations between the intake of dietary fiber and formic acid concentrations in breast milk and the intake of nuts and seeds with propionic acid in breast milk. To the contrary, consumption of refined flour products (i.e., cookies, white pasta, and low-fiber grains) correlated positively with the plasma concentration of butyric, valeric, and caproic acid. A lack of effect of dietary fiber on the fecal abundance of SCFAs has previously been observed in a human intervention study,^71^ despite observed changes in gut microbiome composition. A limitation of our study is that maternal diet was measured only once, reflecting the average consumption over the previous month.

SCFAs have several immunoregulatory functions in vitro and in animal experiments which may reduce the risk of developing atopic disease,^18^^,^^21^, ^22^, ^23^ and higher fecal concentrations of certain SCFAs in infants and young children have been linked to a lower risk of subsequently becoming allergic in several studies.^12^^,^^24^, ^25^, ^26^, ^27^^,^^72^ Here, we found that sensitisation to one or more common allergens measured at 12 months of age correlated negatively with plasma concentrations of several SCFA, namely formic, succinic, isobutyric, valeric, and caproic acid at four months of age. Three of these, formic, succinic and caproic acid were significant also using logistic regression in both unadjusted models and after control for maternal allergy.

Similarly, having atopic eczema at 12 months of age was associated with lower concentrations of acetic, succinic, and isobutyric acid, acetic acid and succinic acid being significant also in a crude logistic regression model, and acetic acid both in a crude and an adjusted model. Food allergy at 12 months was negatively associated with acetic and succinic acid and a tendency towards lower concentrations of valeric acid and total SCFAs, all compared to infants who were non-sensitised and showed no atopic manifestation at 12 months of age. Neither of these associations were, however, significant in logistic regression models.

Part of these associations could be due to reverse causation, as it is possible that parents of allergic children postpone the introduction of new foods and thereby slow the microbiota maturation process; as food allergy and atopic eczema may, in some cases, present already before four months of age. Furthermore, maternal allergy might also be involved as allergic mothers had lower plasma concentrations of succinic acid than non-allergic mothers. However, acetic, butyric, valeric, and caproic acid were not lower in the plasma of allergic than non-allergic mothers, and no difference in effect size was seen when the associations between infant SCFA plasma concentrations and allergy were controlled for maternal allergy status. Still, we cannot rule out that the associations are due to residual confounding.

Further, we could not find any association between SCFA concentrations in breast milk and allergy development in the offspring. To our knowledge, only one previous cross-sectional study has analysed two SCFAs (acetic and butyric acid) in breast milk in relation to atopy in infants and found a negative association between acetic acid concentrations in breast milk and atopic dermatitis severity at two to four months of age.^27^

Milk from atopic mothers contains lower concentrations of acetic and butyric acid than milk from non-atopic mothers.^34^ This is partly in line with our findings of a tendency towards lower butyric acid concentrations and significantly lower caproic acid concentrations in breast milk from mothers with atopic eczema. As caproic and butyric acid were the two SCFAs that were most actively enriched in maternal milk, it is tempting to speculate that maternal atopy could be related to impaired capacity to transport crucial SCFAs into the breast milk, a possibility that can be tested in future studies.

A major strength of this study is the collection of infant plasma together with the corresponding mother’s breast milk and plasma, enabling the investigation of their relations. Another strength is the advanced analytical method using UPLC-MS, which allowed us to detect a large range of SCFAs with high sensitivity.^42^ Also, all children met with the same paediatrician specialised in allergy, and all diagnoses were based on strict, preset criteria. A limitation is that not all maternal plasma samples, and none of the infant plasma samples, were collected under fasting conditions. Further, food intake was measured as an average of the previous month and not in close juncture with the sampling, which should be more informative when investigating concentrations in plasma. However, the questionnaire aimed to capture food habits and it is likely that the intake of several of the food items are kept quite constant over time.

Future studies should correlate SCFA concentrations in faeces and plasma of the same individuals to determine if plasma SCFA are chiefly derived from uptake of colonic SCFAs produced by fermentation of anaerobic bacteria. Investigation of relation between paternal and infant SCFA pattern should reveal the influence of genetics on plasma SCFA concentrations. The negative relations between the concentrations of several SCFAs in infants’ blood plasma and subsequent development of sensitisation and allergy should be followed up at a later time-point and a larger group of allergic children. Nevertheless, the association between low SCFA concentrations and future atopy adds to the evidence of SCFAs as potential immune modulators possibly contributing to healthy tolerance development.

In summary, infants who were either sensitised or had atopic disease at 12 months of age had lower concentrations of some SCFAs in their plasma at four months of age than non-sensitised and non-allergic infants, adding to the evidence of SCFA as potential immune modulators possibly contributing to healthy tolerance development. The concentrations of most SCFAs were lower in breast milk than in maternal plasma, while butyric acid, isobutyric acid, valeric acid, and caproic acid showed higher concentrations and thus seemed enriched in mother's milk, suggesting that the latter might be actively transported from the plasma to the mammary gland. SCFA concentrations in infants’ plasma correlated strongly with SCFAs in maternal plasma but weakly with SCFAs in mothers’ milk.

## Contributors

Malin Barman: Conceptualization, data curation, formal analysis, investigation, project administration, supervision, writing—review and editing.

Monica Gio-Batta: Formal analysis, visualisation, writing—review and editing.

Léna Andrieux: Data curation, formal analysis, investigation, visualisation, writing—original draft.

Mia Stråvik: Data curation, formal analysis, investigation, visualisation, writing—review and editing.

Robert Saalman: Methodology, writing—review and editing.

Rikard Fristedt: Methodology, writing—review and editing.

Hardis Rabe: Investigation, writing—review and editing.

Anna Sandin: Data curation, funding acquisition, methodology, investigation, resources, writing—review and editing.

Agnes E. Wold: Funding acquisition, writing—review and editing.

Ann-Sofie Sandberg: Conceptualisation, methodology, funding acquisition, project administration, supervision, writing—review and editing.

All authors read and approved the final version of the manuscript.

## Data sharing statement

The data presented in this study are not publicly available due to ethical restrictions because they relate to information that could compromise research participant privacy or consent.

## Declaration of interests

The authors declare that they have no known competing financial interests or personal relationships that could have appeared to influence the work reported in this paper.
