# Supplementary material for: Short-chain fatty acids (SCFA) in infants’ plasma and corresponding mother’s milk and plasma in relation to subsequent sensitisation and atopic disease
Source: eBioMedicine. 2024 Feb 9;101:104999. doi: 10.1016/j.ebiom.2024.104999 (PMC10869761; doi:10.1016/j.ebiom.2024.104999)
Supplement: Supplemental Tables S1–S6 [file mmc1.docx]

# ***Supplementary material***

# **Short-chain fatty acids (SCFA) in infants’ plasma and corresponding mother’s milk and**

# **plasma in relation to subsequent sensitisation and atopic disease**

Malin Barman ^1^, Monica Gio-Batta ^2^, Léna Andrieux ^1,3^, Mia Stråvik ^1^, Robert Saalman ^4^, Rikard Fristedt ^1^, Hardis Rabe ^2^, Anna Sandin ^5^, Agnes E. Wold ^2^ and Ann-Sofie Sandberg ^1^

^1^ Department of Life Sciences, Food and Nutrition Science, Chalmers University of Technology, 412 96 Gothenburg, Sweden

^2^ Institute of Biomedicine, Department of Infectious Diseases, University of Gothenburg, 405 30 Gothenburg, Sweden

^3^ Département de Biologie, École Normale Supérieure de Lyon, Université Claude Bernard Lyon 1, 69342 Lyon Cedex 07, France

^4^ Institute of Clinical Sciences, Department of Pediatrics, University of Gothenburg, 405 30 Gothenburg, Sweden

^5^ Department of Clinical Science, Pediatrics, Sunderby Research Unit, Umeå University, 901 87 Umeå, Sweden

Corresponding author: Malin Barman, Chalmers University of Technology, Department of Life Sciences, Food and Nutrition Science, SE-412 96 Göteborg, Sweden. malin.barman@chalmers.se.

**Supplemental Table 1.** Concentration (µmol/L) of short-chain fatty acids in breast milk, maternal plasma, and infant plasma.

| SCFA | Number of carbon atoms | Concentrations | | |  | Ratios | | |
| --- | --- | --- | --- | --- | --- | --- | --- | --- |
|  |  | Infant plasma (N=148) | Maternal plasma (N=142) | Breast milk (N=128) |  | Infant/ maternal plasma | Infant plasma/ breast milk | Breast milk/ maternal plasma |
|  |  | Median (min-max) | | |  | Median (25th-75th percentile) | | |
| Formic acid | 1 | 710 (150-1300) | 460 (100-1300) | 120 (52-230) |  | 1.7 (1.2-2.4) | 5.9 (4.3-7.3) | 0.32 (0.20-0.46) |
| Acetic acid | 2 | 97 (35-320) | 60 (12-240) | 10 (1.3-58) |  | 1.6 (0.96-2.6) | 10 (5.5-17) | 0.18 (0.089-0.29) |
| Propionic acid | 3 | 8.3 (0.00-150) | 5.0 (0.00-200) | 0.10 (0.00-2.0) |  | 1.3 (0.71-1.9) | 160 (25-34000) | 0.0044 (0.000-0.050) |
| Butyric acid | 4 | 0.43 (0.00-3.0 | 0.54 (0.01-2.8) | 61 (7.9-470) |  | 0.64 (0.35-2.1) | 0.0075 (0.0019-0.017) | 100 (46-270) |
| Isobutyric acid | 4 | 0.18 (0.00-2.4 | 0.20 (0.00-1.0) | 0.88 (0.18-7.7) |  | 0.78 (0.49-1.4) | 0.19 (0.11-0.42) | 4.6 (2.3-7.2) |
| Succinic acid | 4 | 4.9 (1.9-15) | 6.4 (2.4-19) | 6.4 (3.8-12) |  | 0.81 (0.65-0.99) | 0.79 (0.58-1.1) | 1.0 (0.72-1.3) |
| Valeric acid | 5 | 0.05 (0.00-0.63) | 0.05 (0.00-0.34) | 0.15 (0.00-1.8) |  | 1.0 (0.44-1.7) | 0.33 (0.04-1.8) | 2.9 (0.67-8.9) |
| Isovaleric acid | 5 | 0.18 (0.00-1.3) | 0.18 (0.0-1.4) | 0.20 (0.0-1.1) |  | 1.00 (0.55-2.11) | 0.9 (0.37-2.1) | 1.1 (0.43-3.4) |
| Caproic acid | 6 | 0.58 (0.09-1.5) | 0.58 (0.09-1.3) | 50 (7.1-350) |  | 0.94 (0.70-1.3) | 0.0094 (0.0042-0.022) | 89 (48-200) |
| Total SCFAs | - | 850 (210-1600) | 550 (160-1300) | 250 (97-1000) |  | 1.6 (1.2-2.3) | 2.96 (1.8-4.6) | 0.64 (0.36-0.92) |

**Supplemental Table 2**. Spearman correlations between short-chain fatty acid concentrations in plasma and breast milk.

|  | **Breast milk - Plasma mother** | |  |  | **Breast milk - Plasma child** | |  |  | **Plasma mother - Plasma child** | |
| --- | --- | --- | --- | --- | --- | --- | --- | --- | --- | --- |
|  | **rho** | **p-value** |  |  | **rho** | **p-value** |  |  | **rho** | **p-value** |
| Formic acid | 0.060 | 0.508 |  |  | 0.107 | 0.228 |  |  | 0.456 | <0.001 |
| Acetic acid | 0.172 | 0.055 |  |  | -0.000 | 0.999 |  |  | 0.200 | 0.017 |
| Propionic acid | 0.158 | 0.079 |  |  | 0.171 | 0.054 |  |  | 0.890 | <0.001 |
| Butyric acid | 0.091 | 0.314 |  |  | 0.035 | 0.693 |  |  | -0.037 | 0.659 |
| Isobutyric acid | -0.074 | 0.412 |  |  | -0.070 | 0.435 |  |  | 0.356 | <0.001 |
| Succinic acid | -0.090 | 0.319 |  |  | 0.038 | 0.670 |  |  | 0.509 | <0.001 |
| Valeric acid | -0.100 | 0.269 |  |  | -0.274 | 0.002 |  |  | 0.563 | <0.001 |
| Isovaleric acid | 0.017 | 0.854 |  |  | -0.134 | 0.131 |  |  | 0.441 | <0.001 |
| Caproic acid | 0.109 | 0.227 |  |  | 0.038 | 0.674 |  |  | 0.489 | <0.001 |

The number of samples: breast milk vs. maternal plasma N=125; breast milk vs. infant plasma, N=128; maternal plasma vs. infant plasma, N=142.

**Supplemental Table 3.** Characteristics for infants with sensitisation, food allergy, atopic eczema, or no allergy.

|  | Non-allergic, non-sensitised  N=109 | Sensitised N=11 | Food allergy N=14 | Atopic eczema  N=19 |
| --- | --- | --- | --- | --- |
|  | n (%) | | | |
| **Sex** |  |  |  |  |
| Boy | 49 (45) | 6 (54) | 8 (57) | 10 (53) |
| Girl | 60 (55) | 5 (46) | 6 (43) | 9 (47) |
| **Firstborn** |  |  |  |  |
| Yes | 56 (51) | 4 (36) | 6 (43) | 9 (47) |
| No | 53 (49) | 7 (64) | 8 (57) | 10 (53) |
| **Mode of delivery** |  |  |  |  |
| Caesarean section | 13 (12) | 1 (9) | 1 (7) | 3 (16) |
| Vaginal delivery | 96 (88) | 10 (90) | 13 (93) | 16 (84) |
| **Pets at home** |  |  |  |  |
| Cat | 27 (25) | 1 (9) | 3 (21) | 3 (16) |
| Dog | 34 (31) | 1 (9) | 2 (14) | 4 (21) |
| Other | 7 (6) | 0 (0) | 1 (7) | 1 (5) |
| **Residential address** |  |  |  |  |
| Town or village | 74 (69) | 9 (82) | 12 (92) | 15 (79) |
| Countryside | 34 (31) | 2 (18) | 1 (8) | 4 (21) |
| **Allergy among family members** |  |  |  |  |
| Mother |  |  |  |  |
| Any allergy | 37 (34) | 6 (55) | 8 (57) | **11 (58)*** |
| Eczema | 9 (8) | 1 (9) | 3 (21) | 2 (11) |
| Food allergy | 11 (19) | 3 (27) | 3 (21) | 4 (21) |
| Asthma | 8 (7) | 3 (27) | **5 (36)**** | 4 (21) |
| Pet allergy | 19 (17) | 3 (27) | 4 (29) | **8 (42)*** |
| Pollen allergy | 27 (25) | 4 (36) | 4 (29) | 7 (37) |
| Father |  |  |  |  |
| Any allergy | 45 (41) | 8 (73) | **10 (71)*** | 10 (53) |
| Eczema | 1 (1) | **2 (18)*** | **2 (14)*** | 2 (11) |
| Food allergy | 15 (14) | 2 (18) | 4 (29) | 2 (11) |
| Asthma | 9 (8) | 1 (9) | 1 (7) | 1 (5) |
| Pet allergy | 28 (26) | **7 (64)*** | **8 (57)*** | 8 (42) |
| Pollen allergy | 30 (28) | **7 (64)*** | 7 (50) | 9 (47) |
| Sibling |  |  |  |  |
| Any allergy | 11 (18) | 2 (29) | 4 (50) | 3 (30) |
| **Allergic comorbidities** |  |  |  |  |
| Sensitised | 0 (0) | - | 9 (64) | 6 (32) |
| Food allergy | 0 (0) | 9 (81) | - | 6 (32) |
| Atopic eczema | 0 (0) | 6 (55) | 6 (43) | - |

Differences in distribution between groups were tested with Fisher’s exact test and Pearson Chi-Square for categorical variables, and Mann-Whitney U test for continuous variables.

**Supplemental Table 4**. Logistic regression results concerning odds of food allergy.

| **Food allergy** | **Unadjusted** | | | **Adjusted^1^** | | |
| --- | --- | --- | --- | --- | --- | --- |
|  | **OR^2^** | **95% CI** | **p** | **OR^2^** | **95% CI** | **p** |
| Formic acid | 0.599 | 0.331-1.086 | 0.092 | 0.629 | 0.347-1.140 | 0.126 |
| Acetic acid | 0.442 | 0.181-1.078 | 0.073 | 0.481 | 0.196-1.185 | 0.112 |
| Propionic acid | 0.310 | 0.307-1.456 | 0.668 | 0.643 | 0.282-1.463 | 0.292 |
| Butyric acid | 1.037 | 0.610-1.762 | 0.894 | 1.085 | 0.638-1.846 | 0.763 |
| Isobutyric acid | 0.630 | 0.199-1.995 | 0.432 | 0.694 | 0.255-1.886 | 0.473 |
| Succinic acid | 0.434 | 0.179-1.056 | 0.066 | 0.466 | 0.193-1.124 | 0.089 |
| Valeric acid | 0.371 | 0.119-1.161 | 0.089 | 0.404 | 0.134-1.217 | 0.107 |
| Isovaleric acid | 1.517 | 0.967-2.380 | 0.070 | 1.426 | 0.897-2.266 | 0.133 |
| Caproic acid | 0.598 | 0.309-1.154 | 0.125 | 0.619 | 0.324-1.182 | 0.147 |

Multiple logistic regression of standardised SCFA concentrations in infant plasma in relation to food allergies at 12 months of age. All models included 14 infants with food allergies and 109 healthy infants. Significant models are marked in bold. Abbreviations: CI—confidence interval, OR—odds ratio.

^1^Adjusted for maternal allergy.

^2^OR per SD of SCFA concentrations, i.e., 244 µmol/L for formic acid, 66 µmol/L for acetic acid, 22 µmol/L for propionic acid, 0,62 µmol/L for butyric acid, 0,28 µmol/L for isobutyric acid, 2,2 µmol/L for succinic acid, 0,11 µmol/L for valeric acid, 0,17 µmol/L for isovaleric acid, and 0,30 µmol/L for caproic acid.

**Supplemental Table 5**. Logistic regression results concerning odds of atopic eczema.

| **Atopic eczema** | **Unadjusted** | | | **Adjusted** | | |
| --- | --- | --- | --- | --- | --- | --- |
|  | **OR** | **95% CI** | **p** | **OR** | **95% CI** | **p** |
| Formic acid | 0.871 | 0.519-1.460 | 0.600 | 0.926 | 0.550-1.560 | 0.772 |
| **Acetic acid** | **0.385** | **0.167-0.884** | **0.024** | **0.421** | **0.178-0.951** | **0.038** |
| Propionic acid | 1.017 | 0.643-1.607 | 0.944 | 1.021 | 0.643-1.621 | 0.931 |
| Butyric acid | 0.935 | 0.571-1.533 | 0.791 | 1.007 | 0.610-1.662 | 0.978 |
| Isobutyric acid | 0.663 | 0.259-1.697 | 0.392 | 0.732 | 0.331-1.622 | 0.443 |
| **Succinic acid** | **0.438** | **0.201-0.956** | **0.038** | 0.481 | 0.220-1.053 | 0.067 |
| Valeric acid | 0.758 | 0.422-1.360 | 0.353 | 0.777 | 0.438-1.380 | 0.389 |
| Isovaleric acid | 1.237 | 0.803-1.906 | 0.334 | 1.154 | 0.742-1.795 | 0.525 |
| Caproic acid | 0.704 | 0.408-1.216 | 0.208 | 0.736 | 0.426-1.271 | 0.272 |

Multiple logistic regression of standardised SCFA concentrations in infant plasma in relation to atopic eczema at 12 months of age. All models included 14 infants with food allergies and 109 healthy infants. Significant models are marked in bold. Abbreviations: CI—confidence interval, OR—odds ratio.

^1^Adjusted for maternal allergy.

^2^OR per SD of SCFA concentrations, i.e., 244 µmol/L for formic acid, 66 µmol/L for acetic acid, 22 µmol/L for propionic acid, 0,62 µmol/L for butyric acid, 0,28 µmol/L for isobutyric acid, 2,2 µmol/L for succinic acid, 0,11 µmol/L for valeric acid, 0,17 µmol/L for isovaleric acid, and 0,30 µmol/L for caproic acid.

**Supplemental Table 6**. Logistic regression results concerning odds of sensitisation.

| **Sensitization** | **Unadjusted** | | | **Adjusted** | | |
| --- | --- | --- | --- | --- | --- | --- |
|  | **OR^1^** | **95% CI** | **p** | **OR^1^** | **95% CI** | **p** |
| Formic acid | **0.394** | **0.179-0.868** | **0.021** | **0.414** | **0.188-0.908** | **0.028** |
| Acetic acid | 0.571 | 0.241-1.355 | 0.204 | 0.619 | 0.258-1.482 | 0.281 |
| Propionic acid | 0.603 | 0.253-1.548 | 0.293 | 0.576 | 0.211-1.573 | 0.282 |
| Butyric acid | 0.900 | 0.468-1.727 | 0.750 | 0.952 | 0.498-1.822 | 0.883 |
| Isobutyric acid | 0.243 | 0.038-1.570 | 0.137 | 0.272 | 0.040-1.862 | 0.185 |
| Succinic acid | **0.173** | **0.044-0.681** | **0.012** | **0.188** | **0.048-0.745** | **0.017** |
| Valeric acid | 0.119 | 0.012-1.137 | 0.065 | 0.133 | 0.014-1.293 | 0.082 |
| Isovaleric acid | 1.311 | 0.792-2.172 | 0.293 | 1.226 | 0.730-2.059 | 0.442 |
| Caproic acid | **0.229** | **0.0.85-0.617** | **0.004** | **0.246** | **0.091-0.664** | **0.006** |

Multiple logistic regression of standardised SCFA concentrations in infant plasma in relation to sensitisation at 12 months of age. All models included 14 infants with food allergies and 109 healthy infants. Significant models are marked in bold. Abbreviations: CI—confidence interval, OR—odds ratio.

^1^Adjusted for maternal allergy.

^2^OR per SD of SCFA concentrations, i.e., 244 µmol/L for formic acid, 66 µmol/L for acetic acid, 22 µmol/L for propionic acid, 0,62 µmol/L for butyric acid, 0,28 µmol/L for isobutyric acid, 2,2 µmol/L for succinic acid, 0,11 µmol/L for valeric acid, 0,17 µmol/L for isovaleric acid, and 0,30 µmol/L for caproic acid.
